# A Perspective on Rabies in the Middle East—Beyond Neglect

**DOI:** 10.3390/vetsci5030067

**Published:** 2018-07-17

**Authors:** Hossein Bannazadeh Baghi, Farbod Alinezhad, Ivan Kuzmin, Charles E. Rupprecht

**Affiliations:** 1 Infectious and Tropical Diseases Research Center, Tabriz University of Medical Sciences, 5166/15731 Tabriz, Iran; 2 Immunology Research Center, Tabriz University of Medical Sciences, 5166/15731 Tabriz, Iran; 3 Department of Microbiology, Faculty of Medicine, Tabriz University of Medical Sciences, 5166/15731 Tabriz, Iran; f4280d@gmail.com; 4 Drug Applied Research Center, Tabriz University of Medical Sciences, 5166/15731 Tabriz, Iran; 5 Medical Branch, University of Texas, Galveston, TX 77555, USA; ivkuzmin@yandex.ru; 6 LYSSA LLC, Atlanta, GA 30301, USA; charles_rupprecht@yahoo.com

**Keywords:** canine vaccination, dog, lyssavirus, Middle East, neglected disease, prophylaxis, rabies, surveillance, zoonosis

## Abstract

Rabies is a neglected but preventable viral zoonosis that poses a substantial threat to public health. In this regard, a global program has been initiated for the elimination of human rabies caused by rabid dogs through the mass vaccination of canine populations. Geographic areas vary greatly towards attainment of this objective. For example, while dog-mediated and wildlife rabies have been largely controlled in major parts of the Americas and Western Europe, the Middle East still grapples with human rabies transmitted by unvaccinated dogs and cats. Rabies prevention and control in the Middle East is quite difficult because the region is transcontinental, encompassing portions of Africa, Asia, and Europe, while consisting of politically, culturally, and economically diverse countries that are often subject to war and unrest. Consequently, one over-riding dilemma is the misinformation or complete lack of rabies surveillance data from this area. This communication is an attempt to provide an overview of rabies in the Middle East, as a cohesive approach for the honing of disease management in each area, based on data compiled from multiple sources. In addition, the related regional transboundary movement of rabies was investigated through phylogenetic studies of available viral gene sequences. Thereafter, the epidemiological status of rabies was assessed for the region. Finally, localities were classified first by the Stepwise Approach towards Rabies Elimination framework and then categorized into four different groups based on management theme: “rabies free”; owned dog and domestic animal vaccination; community dog vaccination; and wildlife vaccination. The classification system proposed herein may serve as a baseline for future efforts. This is especially important due to the severe lack of rabies information available for the Middle East as a whole and a need for a comprehensive program focusing on the entirety of the region in light of renewed international commitment towards canine rabies elimination.

## 1. Introduction

### 1.1. Rabies Situation

Rabies has the highest case fatality rate of any infectious disease and poses an important threat to veterinary and public health, albeit being preventable. Today, this neglected viral zoonosis is present in at least 150 countries and on every continent except for Antarctica. Rabies virus is the most important member of the *Lyssavirus* genus. All warm-blooded vertebrates are susceptible, but the major reservoirs are carnivores and bats. The primary global reservoir and vector for the classical rabies virus that causes rabies in humans is the domestic dog. Currently, rabies has been controlled in canine populations in the Americas and Europe [1]. However, this acute progressive viral encephalitis still wreaks havoc in the majority of Africa, Asia, and the Middle East, where unvaccinated dogs and cats are common. Lyssaviruses are transmitted through the saliva and infect through either a wound or mucosa contamination [2,3]. Whereas human deaths caused by rabies are uncommon in developed countries, due in part to adequate postexposure prophylaxis (PEP), most localities in the Middle East report human rabies cases every year, even though routine surveillance and documentation of definitive cases are extremely unsystematic [4].

The Middle East consists of countries that are politically, culturally, and economically diverse and are often subject to war and unrest. Importantly, a lack of suitable information is rampant about infectious diseases in the area. Misinformation for both civilians and governmental groups can have fatal consequences. Unfortunately, some countries in the Middle East still implement strategies, such as the mass culling of community dogs, rather than investing in canine vaccination campaigns. While this might seem useful at first to local authorities, such tactics been proven ineffective in most instances since these actions actually create an ecological vacuum and draw more animals to the area with newly found high biotic potential based upon local resources [5]. Moreover, the extent of transboundary movement of disease is under appreciated. While close cooperation between neighboring countries could prevent this type of translocation, Middle Eastern neighbors are not always on the best of terms, which makes working together on impending health threats, such as rabies, difficult.

In general, data on rabies in Middle Eastern countries remain poor and inconsistent. Studies that do exist often take a case-by-case approach only, which does not provide adequate insight into the overall epidemiological situation in the area. This communication aims to provide an overview of rabies in the Middle East as one cohesive approach for the refinement of prevention and control methods for the elimination of canine rabies in the region, in keeping with renewed international attention by the Food and Agriculture Organization (FAO) the World Organization for Animal Health (OIE), and the World Health Organization (WHO).

### 1.2. General Descriptive Epidemiology

Rabies is one of the 17 major neglected global tropical diseases and is endemic in most countries. At present, the world can be divided into three principle areas related to rabies: places with enzootic canine rabies; places that have problems with wildlife rabies but canine rabies is under control or has been eliminated; and so-called “rabies-free” countries [6]. Enzootic canine rabies is still at large in most countries of Asia, Africa, and the Middle East. In these regions, people are at risk of both dog-mediated and wildlife-transmitted rabies. Western Europe, Canada, and the United States fall under the second category and, in these areas, transmission through dogs and other canids such as wolves, foxes, jackals, coyotes, etc. has been eliminated or has been controlled. However, rabies can be contracted from wildlife, such as bats, in these and other areas. Presently, rabies has been eliminated fully only in a limited number of regions, which is probably because the majority of these places are islands. For example, Japan, Bahrain, Hawaii, New Zealand, and most of Oceania constitute rabies-free regions. However, rabies-free zones are generally defined as a region that: has not confirmed a case of autochthonous infection brought on by any type of lyssavirus in humans or in animals (including bats) in at least two consecutive years; offers a satisfactory surveillance system; and has an efficiently managed import policy [3]. It should also be noted that even islands are not immune to rabies introduction by imported animals, since there have been instances of rabies outbreaks in previously “free” places like Bali [7] or newly appreciated wildlife reservoirs in locations such as Taiwan [8].

Based on this knowledge, three different rabies patterns were identified for the different animal species that are considered to be the main reservoirs and vectors in an area [6]. An urban rabies cycle is by far the most severe and is present in Africa, Asia, the Middle East, and parts of the New World, such as Bolivia and Haiti. Here, the dog is the most prominent viral reservoir because unvaccinated community dogs are plentiful. A sylvatic (wildlife) cycle occurs when wild carnivores such as jackals, coyotes, mongooses, foxes, etc. are the main or intermediary viral reservoirs. Conversion from this stage to an urban rabies cycle can occur due to interaction among such wildlife and dogs, provided that the dogs are unvaccinated. This example is the principal form in parts of Europe and North America. Another prominent cycle involves bats. Spillover to humans and domestic animals, although not very common, happens mostly from rabid vampire bats and is common in Latin America from Mexico to Argentina [9]. Additionally, in the Americas, distinct insectivorous and frugivorous bat rabies virus variants cause occasional cases in humans, domestic animals, and wildlife. Other lyssaviruses perpetuate among bat populations in Australia, Africa, and Eurasia. Global canine rabies elimination will have no impact upon bat rabies occurrence and subsequent human cases [9]. However, this is not that big of a concern since human rabies caused by non-rabies virus constitutes less than 1% of all worldwide human rabies cases [10].

The majority of Middle Eastern countries fall into the enzootic canine rabies category and are characterized by the presence of both urban and sylvatic rabies cycles. Control methods for animal rabies differ based upon several variables, largely according to knowledge base, stakeholder involvement, cost [4], the enforcement of boundaries, the willingness to vaccinate domestic and community dogs (or mandatory policies for that), and the effectiveness of local surveillance methods. One of the main problems that plagues the Middle East is the cross-boundary movement of animals that might be infected and incubating rabies virus. The majority of animal rabies cases are reported in this region in dogs, foxes, golden jackals (*Canis aureus*), and wolves (*Canis lupus lupus*).

### 1.3. Stepwise Approach towards Rabies Elimination

The aim of this paper is to give an overall assessment of the rabies situation in the Middle East based on the information that is available at this point in time. The objective is based on the fact that information and documentation of the control, prevention, and elimination efforts are key to actually developing a strategy to combat rabies. However, since the Middle East faces constant political, environmental, and cultural upheaval, it is believed that establishing a baseline of information for this area is of the utmost importance for effective control efforts in the future.

The Stepwise Approach towards Rabies Elimination (SARE) framework is a planning and evaluation tool developed for countries in order to develop their activities and to assess their progress towards suitable rabies prevention, control, and elimination. The framework especially focuses on dog-to-human transmitted rabies. Although the SARE framework is used mainly for self-assessment and as a practical guide on a national level, it is the most overarching framework to assess the rabies situation on a larger scale that is accessible today and that is updated regularly. The advantage of using SARE on a national level is that it provides a set of objectives at each stage that must be fulfilled in order to develop to the next stage. This gives countries concrete steps and aims that need to be met and should therefore be able to speed up the rabies elimination process while implementing a One Health approach. SARE focuses on clear lines of communication, a logically flowing chain of commands, and periodical evaluation [11].

## 2. Methods

In this study, the following geographical locations, linking Africa, Europe, and Central Asia, were included subjectively as part of the Middle Eastern region: Bahrain, Cyprus, Egypt, Iran, Iraq, Israel, Jordan, Kuwait, Lebanon, Oman, Palestine, Qatar, Saudi Arabia, Syria, Turkey, the United Arab Emirates, and Yemen. The different localities of the Middle East discussed in this paper were organized according to the SARE framework. The rabies situation was then assessed to see if the SARE framework was sufficient and the regions were then ranked according to their obligation for rabies vaccination and whether rabies is endemic in that region or not.

Data for this review were identified by searches of PubMed, Google, and references from relevant articles and books, using the keywords “Rabies”, “Middle East”, and “the name of respective country”. No date limits were set.

The information included about rabies in each locality was summarized according to the latest accessible scientific data in the public domain, information provided to OIE (http://www.oie.int/animal-health-in-the-world/rabies-portal/) and WHO (http://www.who.int/en/news-room/fact-sheets/detail/rabies), and from communications available at regional scientific conferences and via NGOs (http://www.meereb.org/about-meereb/), focusing primarily upon the past five years.

In addition, to compare viral diversity in the region in light of the above descriptive data, a phylogenetic tree was constructed. Briefly, rabies virus nucleoprotein gene sequences from the Middle East and adjacent geographical areas were obtained from GenBank (Table 1). The sequences were aligned in the Mega 7 program and truncated to 360 nucleotides so that no gaps were present in the alignment. The same program was used for a phylogenetic analysis performed by the neighbor-joining method, based on p-distances between sequences and assuming gamma-distributed substitution rates among sites. Robustness of the analysis was tested in 1000 bootstrap replicates.

## 3. Results

### 3.1. Stage 0

This is the lowest level of the SARE framework and constitutes countries in which there is very little or no information available but where rabies is suspected to be present. This suspicion can be based on episodic clinical descriptions in animals or humans and/or historic confirmations.

#### 3.1.1. Palestine

There is little information available for Palestine where the last human case was reported in 1995 [12].

#### 3.1.2. Syria

Very little information about Syria is accessible, especially after the considerable unrest experienced in the country. It is known that rabies has been endemic in Syria and that it has a higher incidence rate than in Lebanon, its neighbor, and that 24 human cases in the period from 1997 to 2002 were reported [13]. In addition, community and stray dogs are considered the main problem. Without regular veterinary services, the population of unvaccinated dogs is expected to increase, exacerbating the rabies situation.

### 3.2. Stage 1

At this stage, the government gets involved in the fight against rabies and assesses the situation and the structures that have been initiated and also the resources that are available. It is also the stage in which data collection takes place and where new and old data on rabies is gathered in at least part of the country. Focus is also put on following up on cases and outbreaks. This all finally leads to short-term action plans being put in place that can include national rabies prevention and control initiatives and strategies with little to no funding being allocated to the cause.

#### 3.2.1. Iraq

The Republic of Iraq lies between Iran, Turkey, Syria, Jordan, Saudi Arabia, and Kuwait and has a small coastline to the Persian Gulf. The country mainly consists of desert, but close to the two major rivers, the Euphrates and the Tigris, fertile land exists. The very first written records of dog domestication and a description of a rabies-like disease that exists today come from Mesopotamia, which corresponds to modern-day Iraq. During the Middle Ages, Baghdad had been established as the center of the Arab world and was known for its advancements in knowledge, especially in the field of medicine. However, the country was and still is experiencing political unrest and upheaval since the fragmentation of the Ottoman Empire. This, coupled with its central position in the Middle East, with several boarders to other countries in which the disease is endemic, helps to explain why human rabies remains endemic and is reported in all of its 18 governorates [14].

However, it is alarming that the incidence of human rabies in Iraq exceeds that of its neighboring countries substantially. In 2009, the estimated incidence of human rabies in Iraq was 0.89 per million persons while it was 0.025 in Turkey and 0.02 in Iran [15]. This is especially worrisome since the increase in reported cases coincided directly with periods of intense conflict from 2003 onwards and a threefold increase in reported cases for Baghdad during the 10-year period of 2001–2010 [14]. Currently, no new data are available on the rabies situation, but a drastic worsening of the situation is expected due to increasing problems with terrorist groups such as ISIS (which applies to Syria as well). The reason for this sort of increase is expected to be a negative side effect of the migration of medical personnel, a decrease in sanitary conditions, increase of the free-roaming dog populations, and a disruption in municipal infrastructures, services, and responsibilities [14]. All of these factors make disease control and surveillance increasingly difficult if not impossible. Traditionally, the control of the dog population in Iraq was greatly dependent on the elimination of free-ranging animals, which has shown to be ineffective in other countries, including Iran [16]. The animal birth control method (ABC) for free-roaming dogs would be a very recommendable strategy for Iraq, including the vaccination of domestic dogs [16].

#### 3.2.2. Oman

There is very little data published on rabies in Oman. The country was thought to be rabies-free until 1990. The first human case of rabies was reported in 1990 from a young schoolboy who was bitten by a fox [17]. From then onwards, the disease spread across the country. Unlike other countries in the Middle East, Oman deals mostly with sylvatic rabies, in which dogs are not believed to be the essential virus reservoir. Dogs in Oman are not kept as pets and their close proximity to humans is discouraged for cultural reasons. A phylogenetic analysis of samples collected during 2014 showed that all rabies viruses were related to each and that foxes were the main reservoir in the country [18]. The number of animal rabies cases is roughly similar across the governorates and the highest percentages of animal bites to humans were from cats (48.3%), dogs (35.2%), foxes (5.2%), and a few other animals [17]. In Oman, it is probably most advisable to focus on the vaccination of wildlife first before tackling vaccination of domestic animals.

#### 3.2.3. Yemen

Yemen is a low-income country that experiences severe inequality, which puts it among countries with low human development [19]. Rabies is endemic in Yemen and most human cases are the result of canine rabies. Although rabies is a notifiable disease in the country, information on the number of human cases is limited and surveillance is ineffective [20]. The Yemeni Ministry of Health and Population reported that there are roughly 30 human rabies cases per year, although more recent estimates suggest that around 220 are infected with rabies annually. In a 2013 study, the main source of human infection in Yemen were domestic dogs (92%) and the main victims of dog bites were children [21]. A high proportion of suspect cases registered in hospitals were found to be rabies positive, with origin mostly from rural areas. The main factors contributing to rabies occurrence included poor waste disposal and season, with a higher incidence during the summer months [21].

### 3.3. Stage 2

To reach the second stage of the SARE framework, a working intersectoral rabies task force must be in place and rabies must be considered a notifiable disease. Nationwide rabies control and prevention strategies must be recognized and funding needs to be available. Additionally, epidemiological data must be available, regularly updated, and assessed. Dog vaccination campaigns are considered as a response to outbreaks. WHO-compliant PEP and rabies awareness campaigns need to be performed regularly and nationwide.

#### 3.3.1. Egypt

Data are very sparse for this country, especially after the “Arab Spring”. From the limited data available, rabies virus has been confirmed in this area for a long time [22]. However, records about the disease disappeared up until the 18th century [22] and only a few reliable resources exist from the beginning of the 20th century, during which time a “Rabies Institute” was set up in Egypt for PEP and rabies diagnosis. During the late 1990s, the average number of human deaths reported to the WHO was ~30–40 per year and 35 for the year 2000 [23]. The main vectors reported in 2000 were dogs, but cats, ruminants, horses and donkeys, rodents, and mongooses were also considered important [22]. From 2000 to 2010, the situation has been stable, with an annual number of 80 human rabies cases reported [15]. The Zoonotic Disease Department of the Ministry of Agriculture and Land Reclamation reports the annual occurrence of community dog or wild canine bites in humans [23]. All attending bitten persons receive PEP. To improve rabies control in Egypt, serious management of community dogs needs to be implemented, such as through a “trap, vaccinate, neuter, and release” (TVNR) method. Currently, more outdated approaches to dog population control are implemented. The TVNR method has already proven to be successful in a pilot study, which was financed and supported by World Animal Protection (https://www.worldanimalprotection.us.org/). However, the national budget did not allow for the extension of this project.

#### 3.3.2. Iran

The Islamic Republic of Iran is situated in the center of Eurasia and borders several countries in which rabies is endemic. Rabies is considered the most important zoonotic disease in Iran and spreads quite far, including the central desert area, with the most affected areas being in the northeast, east, and south of the country [15]. Although the disease has been a longstanding problem within the area, the very first Pasteur Institute was established in Tehran in 1924. Two years after its establishment, PEP (with the vaccine prepared from dried rabbit spinal cord) was introduced in the country, but only in 1976 were patients vaccinated effectively by a new vaccine which was produced in a human diploid cell culture system [16].

At present, the disease is endemic in both wildlife and domestic animals, with an incidence in humans from 0.02 to 0.05 per million [15,24]. In 2011, rabies was recognized and confirmed in 297 animals, mostly in cattle [25]. The main sources of the disease are stray dogs and wolves, and the disease kills two to six people annually [25]. Serious underreporting is taking place, which affects the improper estimations. Although the government covers PEP expenses completely and has set up approximately 700 health centers in 31 provinces that are open round the clock, many people in rural areas are still not aware of the risks [16]. The country spends a large percentage of its health budget on the increasing demand of rabies PEP, with 80% of all exposure through bites from owned dogs. This is why the Iranian Veterinary Organization is responsible for the vaccination of domestic dogs [16]. However, not all owned dogs are vaccinated, since it is not obligatory, meaning that out of an estimated 900,000 owned dogs, only ~45% are vaccinated [16]. A first step for Iran should be to ensure full vaccination coverage of domestic dogs and, after this is established, the program should extend vaccination coverage to community dogs. These efforts would decrease the rabies threat substantially and would be more lucrative financially in the long term [26]. This is especially important since the removal of free-ranging dogs has proven to be unsuccessful due to spillover from other countries or the repopulation of the areas in which the elimination had taken place by naïve dogs [5].

#### 3.3.3. Turkey

Turkey is distinct in the region with a bridge between the European and Asian continents. The country has a climate that varies greatly and offers diverse habitats for several species of domestic animals and wildlife. Rabies is a known disease, with records dating back to 1887 [12]. Traditionally, rabies was more prevalent in larger cities such as Istanbul [12], but more recent data shows an increase in rabies cases in East Anatolia [27]. This new trend is believed to be related to greater animal industry and lower socioeconomic levels in the east of the country [27]. However, an improvement in surveillance and reporting systems in these areas may have also played a role in the increase of reported cases. Human rabies generally originates from dog bites since dogs are the main host of the virus in Turkey. However, from 1990, foxes have been implicated as a second vector due to spill over from dogs [24], with further independent circulation in fox populations and a host shift from dogs to foxes [27]. Nevertheless, dogs remain the main rabies vector for human disease. One study in 2015 found that among all human rabies cases reported to hospitals, 85% were transmitted by dogs. Of these, 45% were from dogs with no owners. Less than 10% of dogs were vaccinated [28].

### 3.4. Stage 3

To develop further unto this stage, national rabies prevention and control efforts need to be endorsed and funded on a larger scale. Most importantly, proof of dog-vaccination campaigns need to be documented and evidence needs to exist that awareness campaigns are being conducted nationwide. There must also be no indication of dog-mediated human rabies deaths for 12 consecutive months.

#### 3.4.1. Kuwait

Based on the US Centre for Disease Control and Prevention travel advisory website, rabies may be found in dogs, bats, and other mammals in the country [29], although no objective data were found to support this assertion. Vaccination of domestic dogs and cats is obligatory [30].

#### 3.4.2. Lebanon

Rabies is a reportable disease in Lebanon. The last two major reports were published in 2000 and 2013, and the latter included the data accumulated during 2001–2012. Dogs are considered to make up to 91% of all bites to humans, which have been reported to be 440 per year in the period of 2001–2012 [13]. However, it is believed that this figure might not truly reflect the actual number of bites since only those present to a physician are reported. Lebanon in general is quite lax with its rabies surveillance system [13]. It is up to the attending physician to report a rabies case to the district representatives, who then report it to the Lebanese Ministry of Public Health. Nevertheless, Lebanon is considered to be a low-incidence country when compared to other countries in the Middle East, since similar annual occurrence rates have been reported during 1990–1999 [13]. However, this does not necessarily imply that there is rabies “stability” in the country. Since vaccination of dogs is not enforced, the country seems to go from a state of near elimination to a sporadic reemergence, which provides a risk of host switching, where a second reservoir species is infected [13] (as is the case in Turkey with foxes [24]). Also, the position between other rabies-endemic countries can easily lead to spillover, which is a serious issue considering that there is no follow-up investigation of offending animals and incomplete PEP administration to humans is common [13].

### 3.5. Stage 4

Within this stage, countries are not allowed to sustain any indigenous dog-transmitted human rabies deaths for an additional 12 months (after stage 3) or withstand indigenously-acquired, dog-transmitted rabies incidence in any species for one year. Additionally, an effective surveillance system must exist, and evidence must be available that effective measures are being undertaken to prevent the re-emergence of rabies. Updated records of rabies epidemiology within the country must also be offered.

#### Jordan

Human rabies is considered a rare occurrence in Jordan. Many people are aware of the disease and of its danger. The risk is considered very low [31]. The main source of the disease is through dogs and jackals. In 2015, three cases were reported in animals, but no human cases were documented. The vaccination of domestic dogs and cats is obligatory [31]. Since 2011, there has been a joint effort with Israel to manage rabies in the area by oral vaccination, using baits distributed via airplanes at the border [31].

### 3.6. Stage 5

The regions in this stage have to have sustained no dog-transmitted diseases unless they were imported. They must also be free of dog rabies for 12 consecutive months and must publicize this on a national level and, if applicable, to regional organizations. While both Saudi Arabia and Israel fit into this stage, Israel has had a recent extreme flare-up of dog-mediated rabies.

#### 3.6.1. Saudi Arabia

The Kingdom of Saudi Arabia (KSA) is the largest country in the Arabian Peninsula, yet there is little information about its rabies situation. During the period 2007–2009, the Saudi Ministry of Health reported more than 11,000 human bite cases [32]. The majority of animal bites were from dogs (50%) and cats (26.7%), with rodents and camels playing a minimal role [32]. A seasonal difference was reported in the occurrence of bites. Nevertheless, according to the most recent data, the Saudi Ministry of Health has reported no human rabies cases for at least 10 years [33].

#### 3.6.2. Israel

Since 2013 rabies was considered to be a low risk for humans in Israel and is one of the few regions in the Middle East, in which the main rabies vectors are not dogs but red foxes (*Vulpes vulpes*) and golden jackals (*Canis aureus*) [34]. Starting from 1956, domestic dogs must be vaccinated according to the law, first at three months of age and then annually. However, since 2009, canine rabies has re-emerged [31]. The reason is believed to be largely due to young animals who only have received a single vaccination based on the established schedule and because of canine rabies spillover from other regions. This highlights the importance for regional cooperation, even in the face of political instability in the area, because rabies is a regional problem that cannot be solved on an isolated country or case-by-case level. Also, sylvatic rabies in Israel is quite notable. Starting from 1998, annual ORV campaigns have been performed to control the disease in foxes and jackals [31].

### 3.7. Rabies Free

These countries are considered free of dog-mediated rabies. Here, a distinction is made between rabies-free countries of the Middle East and stage 5 countries. This distinction is based on the fact that rabies-free countries in the Middle East are not necessarily so due to their rabies control efforts but because of their geographical location. All rabies-free countries here are either islands or half islands, sharing one land border with another Middle Eastern country.

#### 3.7.1. Bahrain

This island near the Arabian Peninsula has released near to no data on rabies. However, from the few data that are accessible, the country is considered to be a rabies-free zone [35].

#### 3.7.2. Cyprus

Cyprus is a small island in the eastern part of the Mediterranean Sea. The country is considered a rabies-free zone. According to Veterinary Department archives [12] and the WHO/OIE Cyprus Rabies report, there has been no reported case of rabies in domestic animals, wildlife, or humans, except for two cases in 1930 when two imported dogs were found to have rabies and were quarantined. Since then, rabies became a notifiable disease in Cyprus, meaning that all imported dogs and cats need to have official veterinary health certificates to be permitted for import (Regulation (EU) No. 576/2013 of the European Parliament and of the Council of 12 June 2013 on the non-commercial movement of pet animals and repealing Regulation (EC) No. 998/2003). Vaccination is compulsory for animals such as dogs, cats, and ferrets when entering Cyprian territory or traveling to other locations where immunization is required. Upon arrival, animals are blood sampled, receive a booster vaccine dose (with an inactivated rabies vaccine), and remain under strict veterinary monitoring for six months. If the animal is found to be at risk of rabies, it is placed under quarantine.

#### 3.7.3. Qatar

This small country in the Arabian Peninsula has only a single land border with Saudi Arabia and otherwise is surrounded by the Persian Gulf. Although it is not an island, its land border helps Qatar to be one of the few rabies-free countries in the Middle East [33].

#### 3.7.4. United Arab Emirates

The UAE is one of the few countries in the Arabian Peninsula that are considered rabies-free [17].

### 3.8. Specific Geographical Insights

#### Phylogenetic Analysis of Rabies Viruses in the Region

**Figure 1:**
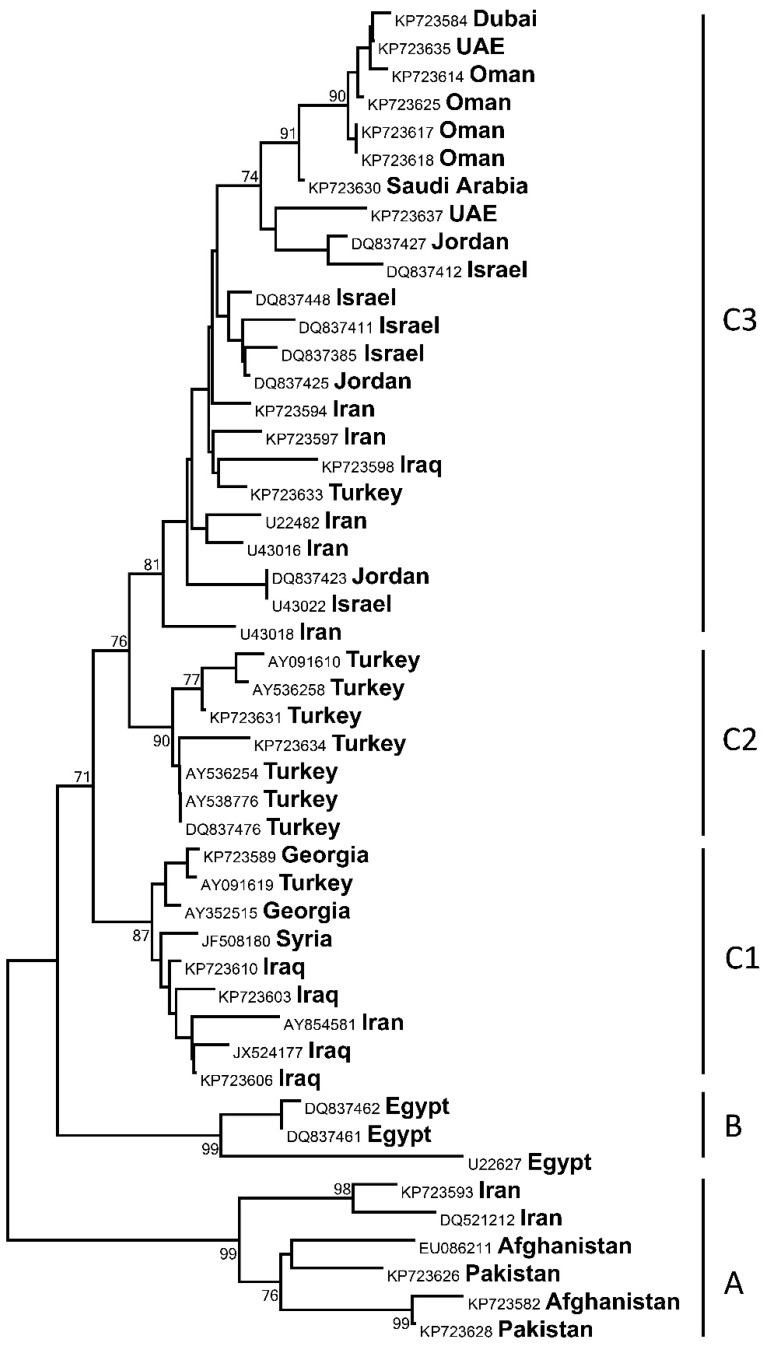
Neighbor-joining phylogenetic tree of sequences (Table 1) included in the present study. Significant bootstrap values (over 70) are shown for the key nodes, and branch lengths are drawn to scale.

Phylogenetic analysis of partial nucleoprotein gene sequences revealed the presence of several phylogenetic lineages of rabies virus in the Middle East (Figure 1). Lineage A was comprised of so-called “Arctic-like” viruses distributed in many areas of Asia, as described in [4]. Among the Middle Eastern viral sequences, only two from Iran were members of this lineage. Lineage B included three available sequences from Egypt. All other sequences belonged to the geographically diverse but genetically homogenous lineage C. Three sublineages segregated within C, with only sublineage C2 showing geographic restriction to Turkey, whereas sublineages C1 and C3 represented a mix of viruses originating from different countries. The sublineages C1, C2, and C3 in this study corresponded to lineages B, C, and D, reported previously [4]. However, as the level of separation (based on genetic heterogeneity) of these lineages is below the level of separation between lineages A, B, and C, and the circulation ranges of viruses overlap significantly, we classify these as sublineages within the lineage C, although each branch may have a separate historical origin [4]. This geographically mixed pattern of lineage C suggests broad movement of virus reservoirs across borders of Middle Eastern countries, which happens either with domestic dogs or wild canids, with a likely host shift occurring between these species, as was demonstrated for Turkey [27].

### 3.9. Category Framework

A variable that was found to be most telling about the rabies situation in Middle Eastern countries was dog vaccinations. It was found that countries that enforced dog vaccinations or in countries where dog vaccinations were mandatory, the country also scored higher on the SARE framework stages.

Operationally, the region could be divided into groups in terms of which steps are needed on the road map to reach the global target of eliminating all human deaths from dog-mediated rabies by 2030 [36].

**Figure 2:**
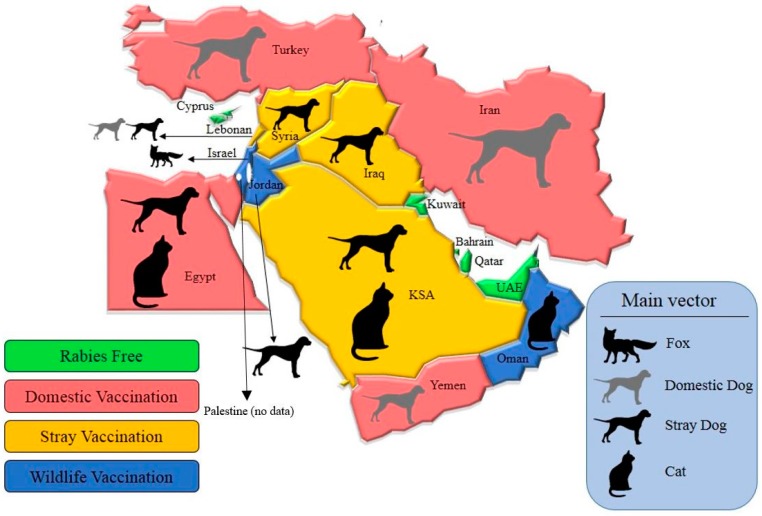
Countries classified into four different rabies groups based on available data.

Based on available data (Table 2), we divided the different areas of the Middle East into four different groups as a first step (Figure 2).

Group 1 consists of countries that are believed to be rabies-free as of the present (with the exception of bat lyssaviruses, for which there has been no information). Here, no major additional steps need to be taken in terms of the elimination of rabies, since most of these territories are “islands”, and as such, new boundary control does not apply, other than what is already in place. However, investing in better laboratory-based surveillance is always advisable since there might be rabies cases that less than ideal surveillance has missed. Group 2 consists of areas in which rabies is endemic and for which the main vector is domestic dogs. In these places, PEP expenses tend to be extremely high, but preventive measures are scarce. The first step to manage rabies more effectively in these nations is to make vaccination of domestic animals compulsory and enforced. At least in Iran and Turkey, public awareness and governmental involvement are both quite high. This could mean that vaccinations can be enforced more easily and would make sense on a long-term financial basis, since PEP expenses could be cut drastically in the end.

The third group includes localities in which rabies is considered low incidence for humans and for which the main transmitters are community dogs. Here, it would make most sense to implement vaccination for such animals and to use a “vaccinate, neuter, and release” method to manage the population of community animals more efficiently.

The last group is made up of places in which a low incidence of human rabies is present, but the main vectors and reservoirs are wildlife. Although Oman is a country in this group, where vaccination of domestic animals is not compulsory, it still has a low human rabies incidence because animal–human contacts are discouraged. Here, dogs are not considered “clean”, so human contact is kept at a minimum. A first step for this group could be to invest more in the vaccination of wildlife (e.g., foxes and jackals), since they are the main source of rabies and can transmit the virus to dogs, other domestic animals, and humans.

A key over riding point that needs to be mentioned is that better surveillance must be implemented in all countries. This preliminary step enables any future interventions to be more effective in relation to time and expenses. The information gathered and the advised strategies for each group are meant to serve as a baseline and to build upon more accessible information. By implementing these first steps, a customized approach to rabies prevention and control can be achieved and aid in the gradual progress towards regional elimination [37]. It is also important to recognize the importance of a transdisciplinary approach within a One Health context, since the success of any program is dependent on collaboration of different organizations from both the human and animal health sectors [38]. Additionally, implementing dog vaccinations on a larger scale within countries through the respective governments, NGOs, and other stakeholders would help not only in the prevention and control of rabies in the country but also effectively raise rabies awareness and might lead to an adherence to the SARE framework and its action plan of development.

## 4. Discussion

The Middle East is comprised of several politically, ethnically, and culturally diverse countries. It houses not only the economies with some of the highest per capita incomes in the world but also severely poverty-stricken nations. Additionally, being a very volatile area makes information gathering and surveillance of any disease, especially internationally neglected diseases, very difficult. This is why a severe lack and inconsistency of such data exists. Rabies, being a global zoonosis in general, seems to be much more neglected in the Middle East than in Africa or Asia, where even informal international groups are able to work on eliminating rabies within the region [20]. This proves to be somewhat more difficult in the Middle East due to constant political and cultural upheaval.

Not only is there a severe lack of information about the rabies situation in different parts of the region, but the information that is accessible tends to be outdated because it does not account for data change during times of war or unrest and might not be correct [33].

Under-reporting is a key issue within the region as a whole, even though rabies is a reportable disease, especially in countries in which the disease is endemic. Rural areas tend to have a higher incidence of human rabies because people living in these areas are more involved in animal husbandry and keep guard animals for security [17]. However, rural people also tend to be less aware of the dangers of rabies, are misinformed about the disease, or simply do not adhere to PEP compliance fully compared to urban residents [13].

Additionally, political instability in the Middle East slows down cooperative efforts immensely. Some areas work together to try and prevent transboundary movement of rabid animals, such as countries that are part of the Middle East and Eastern Europe Rabies Expert Bureau (e.g., Egypt, Iraq, Iran and Turkey) and other parts that have joint programs, such as Jordan and Israel [14]. Nevertheless, rabies prevention is still viewed largely as a local or national approach, if at all. There is no one-size-fits-all approach for the different parts of the Middle East. Taking into account the diversity as well as the socioeconomic and cultural contrasts of the region, an approach to elimination of rabies must be just as complex (Table 1).

Here, the different regions of the Middle East were assessed on the basis of the SARE framework. While this is a helpful first step to analyze the rabies situation in the different localities, it is not sufficient as a standalone rabies assessment and evaluation tool for this part of the world due to its severe lack of consistent information. The SARE framework is also built on the premise that the countries to which this framework is applied are actively pursuing a development in rabies efforts, which obviously cannot apply to war-torn countries of the Middle East such as Syria and Iraq or those that are even facing war-related starvation like Yemen.

The category framework proved to be somewhat related not only to the SARE framework but also to whether rabies was endemic in the country or not. This highlights the importance of dog vaccinations in the fight against dog-mediated rabies and would need further research to strengthen the relation suggested in this paper. Nevertheless, it is important to mention that the paper also faces a very important limitation, namely, the lack of consistent data on rabies from all the regions included in this paper. However, this paper aimed to create a foundation of data for future research in the Middle East.

**Figure 3:**
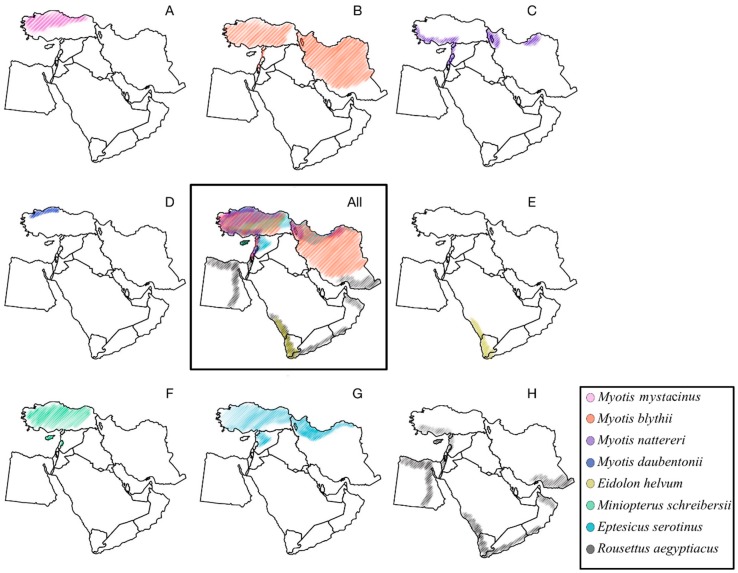
Middle Eastern ranges of bat taxa known as lyssavirus reservoirs in other geographic locations. (**A**) *Myotis mystacinus*; (**B**) *Myotis blythii*; (**C**) *Myotis nattereri*; (**D**) *Myotis daubentonii*; (**E**) *Eidolon helvum*; (**F**) *Miniopterus schreibersii*; (**G**) *Eptesicus serotinus*; (**H**) *Rousettus aegyptiacus*. Compiled from data represented at the web site of IUCN Red List of Threatened Species (http://www.iucnredlist.org).

Unlike attempts at pathogen discovery and characterization elsewhere, only rabies virus has been described in the region, even though several bat species known as lyssavirus reservoirs are present in the Middle East (Figure 3). These include *Myotis blythii* (isolation of Aravan virus reported from Central Asia), *M. mystacinus* (isolation of Khujand virus reported from Central Asia), *M. daubentonii* (the major reservoir of European bat lyssavirus, type 2 in Europe), *M. nattereri* (isolation of Bokeloh bat lyssavirus reported from Europe), *Eptesicus serotinus* (the major reservoir of European bat lyssavirus, type 1 in Europe), *Miniopterus schreibersii* (isolation of West Caucasian bat virus reported from the Caucasus region, and isolation of Lleida bat lyssavirus reported from Spain), and pteropodid bats *Eidolon helvum* and *Rousettus aegyptiacus* (the major reservoirs of Lagos bat virus in sub-Saharan Africa). Given the volatile and migratory character of bats, distribution of bat-borne pathogens usually corresponds to the host species range. As such, all viruses mentioned above are expected to be present in certain areas of the Middle East. Moreover, as surveillance for bats for pathogens in the Middle East is quite limited, other lyssaviruses may be present in these or other bat species. Additional significance of bat lyssaviruses for veterinary and public health is caused by their genetic and antigenic differences from rabies virus, used for production of rabies biologics. For example, rabies biologics do not confer protection against West Caucasian bat virus and Lagos bat virus (the same is expected for Lleida bat lyssavirus given its phylogenetic relationships within the *Lyssavirus* genus).

Notwithstanding the probability of other lyssaviruses, from medieval times to the 21st century, the implicit role of the dog in rabies epidemiology has been appreciated throughout the region [39,40]. Now, a reinvigorated strategy is needed within the Middle East, be it countries still grappling mainly with dog-transmitted rabies or those in which wildlife species are the reservoirs of the disease, to implement a customized action plan to manage this zoonosis successfully and to take effective steps towards the global FAO, OIE, and WHO rabies goal of human rabies elimination by 2030.

## 5. Conclusions

Rabies, and especially dog-mediated rabies, remains a threat to the entirety of the Middle East. While not all regions within the Middle East suffer from direct disadvantages, such as human deaths, the threat of rabies is and will remain ever looming as long as neighboring regions suffer from rabies. The review highlighted mainly that the data that is available today is in no way sufficient and that more research must be performed in this region. It was also found that generic frameworks, such as the SARE framework, are not perfectly applicable in the categorization of different regions of the Middle East based on the rabies threat. Rather, it was found that a better indicator of the rabies situation and elimination efforts was the state of dog vaccinations. If dog-vaccinations were obligatory then the region scored higher also on the SARE framework. This, it is believed, highlights the importance of dog-vaccinations in light of rabies elimination efforts. Therefore, making dog-vaccinations obligatory could be the key to fighting dog-mediated rabies more efficiently in the Middle East. 

## Figures and Tables

**Table 1:** Rabies virus gene sequences used in this study.

Origin	Year	Genbank #	ID	Species	Details/Reference
Afghanistan	2004	EU086211	04029AFG	dog	Bourhy et al., 2008
Afghanistan	2009	KP723582	20516	dog	Horton et al., 2015
Dubai	2013	KP723584	30440	fox	Horton et al., 2015
Egypt	1979	U22627	na	human	Kissi et al., 1995
Egypt	1998	DQ837461	na	dog	David et al., 2009
Egypt	1998	DQ837462	na	dog	David et al., 2009
Georgia	1989	AY352515	RV305	dog	Kuzmin et al., 2004
Georgia	2014	KP723589	160	na	Horton et al., 2015
Iran	2000	AY854581	V686	cow	Nadin-Davis et al., 2003
Iran	2000	DQ521212	na	sheep	Nadin-Davis et al., Direct submission
Iran	1991	KP723593	13158	wolf	Horton et al., 2015
Iran	1991	KP723594	13159	wolf	Horton et al., 2015
Iran	1991	KP723597	13164	hyena	Horton et al., 2015
Iran	1986	U22482	8681IRA	dog	Kissi et al., 1995
Iran	1993	U43016	9308IRA	jackal	Bourhy et al., 1999
Iran	1993	U43018	9320IRA	wolf	Bourhy et al., 1999
Iraq	2011	JX5241177	RV2517	dog	Horton et al., 2013
Iraq	2004	KP723598	20276	dog	Horton et al., 2015
Iraq	2007	KP723603	20286	dog	Horton et al., 2015
Iraq	2008	KP723606	20291	dog	Horton et al., 2015
Iraq	2009	KP723610	20297	dog	Horton et al., 2015
Israel	2004	DQ837385	na	fox	David et al., 2009
Israel	2000	DQ837412	na	fox	David et al., 2009
Israel	1998	DQ837411	na	fox	David et al., 2009
Israel	1998	DQ837448	na	dog	David et al., 2009
Israel	1993	U43022	9332ISR	jackal	Bourhy et al., 1999
Jordan	1999	DQ837423	donkey/J2/1999	donkey	David et al., 2009
Jordan	1998	DQ837425	cow/J4/1998	cow	David et al., 2009
Jordan	1998	DQ837427	badger/J6/1998	badger	David et al., 2009
Oman	1991	KP723614	13145	na	Horton et al., 2015
Oman	2002	KP723617	RVI	Fox	Horton et al., 2015
Oman	2002	KP723618	RVII	fox	Horton et al., 2015
Oman	2004	KP723625	RVXI	camel	Horton et al., 2015
Pakistan	1979	KP723626	13088	dog	Horton et al., 2015
Pakistan	1989	KP723628	RV195	dog	Horton et al., 2015
Saudi Arabia	1990	KP723630	13044	fox	Horton et al., 2015
Syria	2010	JF508180	na	wolf	Johnson et al., direct submission
Turkey	2001	AY091610	RV1124	fox	Johnson et al., 2003
Turkey	2001	AY091619	RV1133	dog	Johnson et al., 2003
Turkey	2003	AY536254	RV1385	jackal	Johnson et al., 2006
Turkey	2003	AY536258	RV1389	fox	Johnson et al., 2006
Turkey	2001	AY538776	RV1142	dog	Johnson et al., 2006
Turkey	2000	DQ837476	T3	dog	David et al., 2009
Turkey	2001	KP723631	RV1138	dog	Horton et al., 2015
Turkey	2000	KP723633	RV1145	fox	Horton et al., 2015
Turkey	2003	KP723634	RV1382	dog	Horton et al., 2015
UAE (Emirates)	1991	KP723635	RVXII	camel	Horton et al., 2015
UAE (Emirates)	1994	KP723637	RVXIV	dog	Horton et al., 2015

na = data not available.

**Table 2:** Comparison of generalized rabies status within the Middle East.

Region	Human Rabies	Compulsory vaccination of domestic animals	Main Vectors	Reservoirs
**Bahrain**	“Free”	n/a	n/a	n/a
**Cyprus**	“Free”	Yes	n/a	n/a
**Egypt**	Endemic	No, but vaccination is available	Community dogs and cats	Community dogs
**Iran**	Endemic	No, but available	Domestic dogs and wolves	Community dogs
**Iraq**	Endemic	Yes	Community dogs	Community dogs
**Israel**	Low-Incidence	Yes	n/a	Foxes, jackals
**Jordan**	Low-Incidence	Yes	Community dogs	Community dogs and jackals
**Kuwait**	“Free”	Yes	n/a	n/a
**Lebanon**	Low-Incidence	No, but vaccination is available	Dogs	Community dogs
**Oman**	Low-Incidence	No	Cats	Foxes
**Palestine**	Low-Incidence	n/a	n/a	n/a
**Qatar**	Free	n/a	n/a	n/a
**Saudi Arabia**	Low-Incidence	Yes	Community dogs and cats	Community dogs
**Syria**	Endemic	No	Community dogs	Community dogs
**Turkey**	Endemic	No, but vaccination is available	Dogs	Foxes and community dogs
**UAE**	“Free”	Yes	n/a	n/a
**Yemen**	Endemic	No	Dogs	Community dogs and wildlife (unknown)

**Key:** 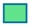
—Group 1 “Rabies-Free” 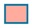
—Group 2 Domestic Dog Vaccination. 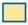
—Group 3 Community Dog Vaccination. 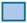
—Group 4 Wildlife Vaccination.
